# Suicidal Ideation and Its Associated Factors in Medical, Dental, and Pharmacy Students: A Cross-Sectional Study during COVID-19 Pandemic

**DOI:** 10.1155/2022/8139351

**Published:** 2022-11-28

**Authors:** Amin Nakhostin-Ansari, Mitra Akhlaghi, Farnaz Etesam, Mohammad Hossein Sadeghian

**Affiliations:** ^1^Sports Medicine Research Center, Tehran University of Medical Sciences, Tehran, Iran; ^2^Department of Forensic Medicine, School of Medicine, Tehran University of Medical Sciences, Tehran, Iran; ^3^Psychosomatic Medicine Research Center, Imam Khomeini Hospital, Tehran University of Medical Sciences, Tehran, Iran

## Abstract

**Objectives:**

This study is aimed at comparing the prevalence of suicidal ideation among Iranian medical, dental, and pharmacy students and determining the demographic and basic characteristics and mental and psychological issues associated with suicidal ideation in these students.

**Methods:**

This cross-sectional online survey was conducted during the 2020-2021 academic year on medical, dental, and pharmacy students studying at the Tehran University of Medical Sciences (TUMS). The questionnaire consisted of six sections: Beck Hopelessness Scale (BHS), General Health Questionnaire (GHQ), Perceived Stress Scale (PSS), UCLA loneliness scale, Maslach Burnout Inventory-Student Survey (MBI-SS), and a questionnaire that was designed to evaluate students' family history, current psychological status, and basic and demographic characteristics.

**Results:**

In total, 419 students participated in our study, with 133 (31.7%) being medical students, 85 (20.3%) being pharmacy students, and 201 (48%) being dental students. In our study, the prevalence of suicidal ideation was 32%. Family history of psychological issues (OR = 2.186, P =0.012), current or past smoking (OR = 2.155, *P* = 0.01), parents not living together (OR = 2.512, *P* = 0.046), and satisfaction with the current field (OR = 0.51, *P* < 0.001) were all independently associated with the presence of suicidal ideation. Also, higher scores in BHS (OR = 1.167, *P* < 0.001), PSS (OR = 1.081, *P* = 0.001), and UCLA loneliness scale (OR = 1.057, *P* < 0.001) were independently associated with a higher risk of suicidal ideation.

**Conclusion:**

The prevalence of suicidal ideation among Iranian medical, dental, and pharmacy students is relatively high and has increased during recent years, which needs emergent action.

## 1. Introduction

Suicide is defined as an individual intentionally ending their own life and is one of the major public health issues worldwide [1, 2]. According to the global burden of disease study, in 2016, 817000 deaths due to suicide were recorded globally, indicating a 6.7% increase from 1990 [3]. In Iran, there has also been a similar increasing trend in the mortality and years of life lost (YLL) rates of suicide, especially in males, in recent years [4]. Even though suicide is a public health concern in all age groups, most suicides occur in individuals aged 20-25, most from low and middle-income countries [1]. Suicide is also the second cause of death in individuals aged 15 to 29 years [1]. Similarly, there has been a shift in the incidence of suicide from middle-aged to younger individuals in Iran, and Iran's highest suicide attempt and suicide mortality rates have been in the 15-24 and 25-34 age groups, respectively, leading to the highest YLL compared to other age groups [4–6].

A suicide attempt is an endpoint of a complex process consisting of three premotivational, motivational, and volitional phases [7–9]. Multiple environmental, biological, and psychological factors (premotivational phase) may lead to suicidal ideation (motivational phase) in an individual; however, the presence of suicidal ideation does not necessarily lead to a suicide attempt (volitional phase), with about 40% of individuals with suicidal ideation do not attempt suicide in a year after the development of these thoughts [8, 10, 11]. Other factors, such as impulsivity, play roles in someone with suicidal ideation acquiring suicide capability and committing suicide [8, 10]. Therefore, screening for suicidal ideation is important as these people benefit from preventive interventions. Also, identifying the risk factors of suicidal ideation and designing interventions to reduce their burden can be strategies to decrease the incidence of suicidal ideation [12, 13]. Interventions improving students' mental health, such as those enhancing resilience and mindfulness, might also reduce the incidence of suicidal ideation [14, 15].

Some people are at higher risk of suicide which may be targeted in suicide prevention programs [16]. Healthcare providers are of these high-risk populations, and pharmacists, dentists, and doctors are among the high-risk occupations for suicide [17]. Studies on the prevalence of suicidal ideation and suicide risk among medical doctors of different countries have indicated a higher risk of suicide in this population compared to the general population [18–24]. In their first year at medical school, the prevalence of psychological issues, which are the main risk factors for suicidal ideation, in medical students is not significantly different from those of the same age [25–27]. However, the situation worsens during medical school education. In the later years of education, the prevalence of suicidal ideation increases in medical students, which may be a reason for the higher risk of suicidal ideation among doctors [23, 28]. Many studies have evaluated suicidal ideation and its possible risk factors among medical students. They have found several mental and psychological factors that may lead to suicidal ideation, including burnout, depression, anxiety, neuroticism, and obsessive-compulsive disorder [19, 28–31]. Affective temperamental dysregulation is also associated with higher levels of hopelessness, which can increase the risk of suicidal ideation [32]. However, despite the high prevalence of suicidal ideation in pharmacy and dental students, even comparable to medical students, there are only limited studies on the prevalence and predictors of suicidal ideation among these students [33–38], warranting more comprehensive studies on these students. It gets more important considering the rise in suicidal ideation among students during the COVID-19 pandemic [39]. The COVID-19 pandemic has put healthcare workers at a higher risk of psychological issues, fatigue, stress, and burnout [40]. For example, about 50% of medical students have experienced burnout during the COVID-19 pandemic, which can be a risk factor for suicidal ideation [41]. These findings warrant further action to address these psychological issues and students in the related fields to prevent further devastating consequences, such as suicidal ideation and suicide [41].

Studies on suicidal ideation in healthcare professions have been very limited in Iran. In two studies evaluating suicidal ideation among medical students, the prevalence of suicidal ideation was estimated to be about 16%, indicating a higher prevalence of suicidal ideation among Iranian medical students than the global average of 11% [29, 42, 43]. These studies also found that hopelessness and neuroticism are associated with higher risks of suicidal ideation among Iranian medical students [42, 43]. In another study in Iran, the prevalence of suicidal ideation among nursing, midwifery, and medical emergency students was about 26% [44].

To date, no study has evaluated the prevalence of suicidal ideation among Iranian dental and pharmacy students. Even on a global scale, there are limited studies on suicidal ideation in these populations. Also, most studies evaluating the psychological issues associated with suicidal ideation have focused on a limited number of factors. Only a few comprehensive studies have evaluated the associations of multiple psychological and mental factors with suicidal ideation in medical students. Therefore, this study was conducted to address three aims. First, determining the prevalence of suicidal ideation among Iranian pharmacy and dental students compared to medical students. Second, determining basic and demographic characteristics associated with suicidal ideation in Iranian medical, pharmacy, and dental students. Third, determining the mental and psychological factors, including hopelessness, stress, loneliness, and burnout, independently associated with suicidal ideation among Iranian medical, pharmacy, and dental students. We hypothesized that the prevalence of suicidal ideation is significantly different among medical, dental, and pharmacy students, and psychological issues are associated with a higher risk of having suicidal ideation among students.

## 2. Methods

### 2.1. Study Design and Setting

This cross-sectional study was conducted during the 2020-2021 academic year on medical, dental, and pharmacy students studying at the Tehran University of Medical Sciences (TUMS). Our study used convenience sampling methods. In the first step, we designed an online questionnaire using Google Forms, which contained questionnaires evaluating students' basic and demographic characteristics, family and past medical and psychological history, hopelessness, loneliness, stress, burnout, and suicidal ideation. In the second step, we drafted a message containing the study goals and objectives, inviting the students to participate in the study if they were interested, and had a link to the online questionnaire. Finally, using convenience sampling, we sent the questionnaire to the available and known medical, pharmacy, and dental students' groups and channels on social media, such as WhatsApp. We also asked them to share the message with their friends and colleague, if possible. Those interested in participating in the study could open the link and complete the questionnaire. The study was conducted according to the Declaration of Helsinki, and participation in the study was voluntary. The ethics committee of TUMS approved the study protocol (ethics code: IR.TUMS.MEDICINE.REC.1397.428).

### 2.2. Participants

Our inclusion criteria were as follows: (1) studying medicine, dentistry, or pharmacy at TUMS during the 2020-2021 academic year, (2) Iranian nationality, (3) understanding the Persian language, and (4) giving consent to participate in the study. Medical residents and students of other healthcare fields were excluded from the study. Also, those students who were not from Iran were excluded. The students were enrolled regardless of their history of psychological diseases, suicide attempts, stage of education, and ethnicity.

### 2.3. Data Collection Tools

The questionnaire consisted of six sections: Beck Hopelessness Scale (BHS), General Health Questionnaire (GHQ), Perceived Stress Scale (PSS), UCLA loneliness scale, Maslach Burnout Inventory-Student Survey (MBI-SS), and a questionnaire that was designed to evaluate students' family history, current psychological status, and basic and demographic characteristics. We selected the questionnaire based on their validated Persian version's availability and use in previous studies evaluating suicidal ideation and psychological issues among students to compare our findings with previous studies.

The first questionnaire included students' family history, current psychological status, and basic and demographic characteristics. It consisted of questions on students' gender, age, the year they entered the university, current residence, marital status, reason for choosing their current field, and whether they had investigated their current field before choosing it. Students were asked to determine how satisfied they were with their current field on a Likert scale from 1 (least satisfaction) to 5 (most satisfaction). The questionnaire also consisted of questions evaluating their family status, including parents' ages and educational status, parents' death, family income per month, and whether their parents are living together at the moment or not. There were also questions evaluating students' and their family history of smoking, addiction, psychological issues, self-harm, and suicide attempt.

#### 2.3.1. The Beck Hopelessness Scale (BHS)

BHS was designed by Beck et al. for the evaluation of hopelessness. It consists of 20 true-false items, and 1 point belongs to the pessimism answer to each item. The total score is the sum of all items' scores and is calculated out of 20, with higher scores indicating more severe hopelessness [45]. The Persian version of BHS was used in the current study, which has high reliability and good internal consistency (Cronbach's alpha of 0.79) in evaluating hopelessness among Iranians [46, 47].

#### 2.3.2. The General Health Questionnaire (GHQ)

The GHQ, designed by Goldberg and Goldberg and Hillier, is a reliable and valid screening tool for psychological issues, consisting of 28 items and four subscales, including social dysfunction, depression, anxiety and insomnia, and somatic symptoms. Each item is scored on a Likert scale from 0 to 3, with higher scores indicating more severe symptoms in each subscale [48, 49]. GHQ has been translated into Persian and is validated for use among Iranians. The Persian version of the GHQ has a Cronbach's alpha of 0.923. Also, Cronbach's alpha value was above 0.7 for all subscales [50].

We used four items from the Persian version of GHQ to evaluate suicidal ideation in the past month. These items were “Thought of the possibility that you might make away with yourself,” “Found that the idea of taking your own life kept coming into your mind,” “Felt that life isn't worth living,” and “Found yourself wishing you were dead and away from it all.” A score of 2 or 3 for any of these items was considered suicidal ideation [43, 48, 49, 51].

#### 2.3.3. The Perceived Stress Scale (PSS)

PSS was designed by Cohen et al. for the evaluation of perceived stress. This questionnaire consists of 14 items that are scored on a Likert scale from 0 to 4. The total score is the sum of all items' scores and is calculated out of 56, with higher scores indicating more severe perceived stress [52]. Our study used the Persian version of PSS, a reliable tool with acceptable internal constancy (Cronbach's alpha = 0.78) for evaluating perceived stress in the past month among Iranian students [53].

#### 2.3.4. The UCLA Loneliness Scale

The UCLA loneliness scale, designed by Russel et al. and Russell DW, is a standard tool for evaluating loneliness. This questionnaire consists of 20 items, with 11 scored on a Likert scale from 1 (never) to 4 (always), and the scoring is reversed for the remaining items. The total score is the sum of all items' scores and ranges from 20 to 80, with higher scores indicating more severe loneliness [54, 55]. Our study used the Persian version of the UCLA loneliness scale, a reliable (Cronbach's alpha = 0.91) and valid tool evaluating loneliness among Iranians, including students [56, 57].

#### 2.3.5. The Maslach Burnout Inventory-Student Survey

The MBI-SS was designed to evaluate burnout among students and is a modified version of the Maslach Burnout Inventory-General Survey. MBI-SS consists of three subscales, exhaustion (five items), cynicism (four items), and professional efficiency (six items). Each item is scored on a Likert scale from 0 to 6. The total score of each subscale is the sum of all related items' scores, and for exhaustion and cynicism, higher scores indicate more burnout. For professional efficiency, lower scores indicate more burnout [58, 59]. We used the Persian version of MBI-SS, a reliable and valid tool for evaluating burnout among Iranian students. All subscales also have a good internal consistency with Cronbach's alpha of higher than 0.8 for all subscales. Also, the test-retest reliability of the Persian version of MBI-SS is approved with a reliability coefficient higher than 0.65 for all subscales [60].

### 2.4. Statistical Analysis

We calculated the mean and standard deviation (SD) for continuous variables and the number and percentage for categorical variables. We used the Chi-square or Fisher's exact tests to compare the categorical variables across groups. Also, the Kolmogorov-Smirnov test was used to determine whether continuous variables are distributed normally or not. As none of these variables were distributed normally (*P* < 0.05), we used nonparametric tests to compare continuous variables across groups, including the Mann-Whitney and Kruskal-Wallis tests. We considered *P* ≤ 0.05 as statistically significant. Two multiple backward stepwise binary logistic regression models were used to determine the basic characteristics associated with suicidal ideation and questionnaire scores independently associated with suicidal ideation. SPSS software (version 22 for windows, SPSS inc., Chicago, Illinois) was used for analyses.

## 3. Results

In total, 419 students participated in our study, with 133 (31.7%) being medical students, 85 (20.3%) being pharmacy students, and 201 (48%) being dental students. In our study, the prevalence of suicidal ideation was 32%, and 18 (4.3%) students had attempted suicide. Forty-five medical students (33.8%), 29 pharmacy students (34.1%), and 60 dental students (29.9%) had suicidal ideation at the time of the study.

Family history and the basic and demographic characteristics of participants are shown in Table 1. Fifty medical students (37.6%), 50 pharmacy students (58.8%), and 117 (58.2%) dental students were female. The mean age of medical students was 24.15 (SD = 5.81), which was significantly higher than the mean age of pharmacy students (mean = 21.35, SD = 2.52, and *P* = 0.001), but was not significantly different with dental students (mean = 21.89, SD = 3.25, and *P* = 0.052). The prevalence of suicidal ideation was not significantly different between males and females (*P* = 0.465). However, the mean age of those with suicidal ideation was significantly higher than those without suicidal ideation (22.88 vs. 22.31, *P* = 0.006). The prevalence of suicidal ideation was significantly lower in those who had never smoked and those without a history of or current addiction (*P* < 0.01).

Psychological issues and suicidal ideation are compared between medical, dental, and pharmacy students in Table 2. BHS score was significantly lower (*P* < 0.05) in dental students (mean = 5.9 and SD = 4.17) than in medical (mean = 7.28 and SD = 4.72) and pharmacy students (mean = 7.8 and SD = 4.59). Also, the mean UCLA loneliness scale score was significantly higher in pharmacy students than in dental students (49.3 vs. 44.23, *P* = 0.007). The prevalence of diagnosed psychological issues was significantly higher in pharmacy students compared to other students (*P* = 0.046). Also, the mean scores of all questionnaires and the prevalence of diagnosed psychological issues were significantly higher in students with suicidal ideation than in those without (*P* < 0.001).

The results of the multiple binary logistic regressions are shown in Table 3. Family history of psychological issues, current or past smoking, parents not living together, and less satisfaction with the current field were all independently associated with a higher risk of having suicidal ideation (*P* < 0.05). Also, higher scores in PSS, UCLA loneliness scale, and BHS were independently associated with a higher risk of suicidal ideation (*P* < 0.05).

## 4. Discussion

We found that about 30% to 35% of these students experienced suicidal ideation at the time of the study, indicating concerning levels of suicidal ideation among them. We found that higher levels of hopelessness, loneliness, and stress are associated with a higher risk of suicidal ideation among students. Also, family history of psychological issues, separation of parents, dissatisfaction with the current field, and history of smoking during life were independently associated with suicidal ideation.

We found that the prevalence of suicidal ideation among Iranian medical, dental, and pharmacy students is 33.8%, 29.9%, and 34.1%, respectively, and there was no significant difference between groups in this regard. Suicidal ideation among Iranian pharmacy and dental students was not evaluated before. However, previous studies on suicidal ideation in Iranian medical students during the 2018-2019 academic year had found a prevalence of about 16% [42, 43], which is considerably lower than our findings in the 2020-2021 academic year. Also, the global average of suicidal ideation among medical students is estimated to be 11%, indicating the current high prevalence of suicidal ideation among Iranian students is worrying, warranting serious and emergency actions [29]. One possible explanation for such an increase in the prevalence of suicidal ideation among Iranian students may be due to conducting the study during the COVID-19 pandemic, which may lead to an increase in suicidal ideation among the population [61, 62]. However, a study on Bangladeshi medical students revealed no increase in the prevalence of suicidal ideation among them during the COVID-19 pandemic compared to the prepandemic situation [39]. Therefore, the COVID-19 pandemic may not justify this considerable increase in the prevalence of suicidal ideation among Iranian students. Also, recent sanctions and the economic crisis during the COVID-19 pandemic may have worsened Iranian people's financial and socioeconomic status [63, 64], which can be another factor leading to psychological issues and subsequent increase in suicidal ideation considering the association between worse socioeconomic status and higher risk of having suicidal ideation [65, 66]. There is still a need for studies to evaluate further the role of possible underlying factors, such as curricula and workload [67, 68]. Also, considering the high prevalence of suicidal ideation, there is a need for actions, such as screening students and interventions targeting modifiable predisposing factors to avoid consequences. Several strategies, such as improving spirituality and implementing preventive measures in both academic and clinical settings, might be beneficial in reducing the incidence of suicidal ideation and psychological issues among students during the pandemic [69].

We found no significant difference in the prevalence of suicidal ideation among medical, dental, and pharmacy students. Few studies are comparing suicidal ideation among students in these fields. In a study in Brazil, Alexandrino-Silva et al. found no significant difference in suicidal ideation among pharmacy and medical students [33]. Similarly, in another study, the prevalence of suicidal ideation among medical and pharmacy students in the United States (US) was 4.7% and 2.2%, respectively, and there was no significant difference between groups in this regard [70]. Alexandrino-Silva et al. hypothesized that unmeasured confounding factors might have led to a lack of difference between groups [33]. However, studying in one of the mentioned fields was not independently associated with suicidal ideation in our regression models. In another study in Saudi Arabia, the prevalence of suicidal ideation in the year before the study was significantly higher in dental students (38%) than medical (29%) students [71]. In contrast, studies in the UK indicated a higher prevalence of suicidal ideation and suicidal attempts among medical students than pharmacy and dental students [72, 73]. Overall, these studies indicate that except in the UK, the prevalence of suicidal ideation is not much different between medical, pharmacy, and dental students, and the prevalence of suicidal ideation in these students is higher than in the general population [73]. The situation also gets more complicated considering healthcare field students have low intention to seek help for their psychological issues, including suicidal ideation [70]. These fields share common stressors and predisposing factors for suicidal ideation, such as high academic expectations from students, performance pressure, heavy workload, information overload, and higher psychological issues, leading to a higher prevalence of suicidal ideation among students compared to the general population [74–78]. In addition, there may be some specific stressors and risk factors for suicidal ideation in each field, that are needed to be determined in future studies, especially in dental and pharmacy students that there are limited studies on specific risk factors of suicidal ideation among them.

We found several characteristics independently associated with a higher risk of suicidal ideation among students, including family history of psychological issues, current or past smoking, and separation of parents, which can be utilized in the programs designed to find students at high risk of suicidal ideation. Smoking, in particular, is a possible risk factor for suicidal ideation in students. Sathian et al. found a dose-response relationship between the average number of cigarettes smoked during the week and the presence of suicidal ideation in young adults [79]. Waters et al. found an association between smoking and suicidal ideation among college students independent of substance or alcohol abuse and major depressive disorders [80]. Therefore, primary preventive programs to prevent students start smoking and smoking cessation interventions may also be beneficial in reducing the prevalence of suicidal ideation among students.

We found that hopelessness, stress, and loneliness are independently associated with increased odds of suicidal ideation among students, while burnout was not an independent factor associated with suicidal ideation. It is one of the strengths of the current study as we took several important factors into account while evaluating the factors associated with suicidal ideation. Our findings align with previous studies, as suicidal ideation's associations with hopelessness, stress, and loneliness were previously evaluated and established among different students [19, 33, 43, 71, 81]. Our findings may have several practical implications. First, symptoms of loneliness, hopelessness, and stress can be used to screen students to determine those at a higher risk of suicidal ideation. Second, the learning environment affects hopelessness and perceived stress among students [82, 83]. Therefore, future studies on characteristics of the learning environment in Iranian universities and their associations with hopelessness and perceived stress may be beneficial to guide future interventions, to optimize the learning environment, and to reduce the stress and hopelessness levels and subsequent suicidal ideation among students. Third, high workloads in the medical, dental, and pharmacy fields in Iran may be a factor leading to stress and subsequent suicidal ideation among the students [84]. This point should be considered in designing these fields' curricula in the future, as adjusting curricula and reducing the workload may benefit students' mental health and decrease the risk of suicidal ideation.

This study had a few limitations. First, this study was cross-sectional, which cannot demonstrate the causal relationship between the variables, and future longitudinal studies are needed. Second, less than 100 pharmacy students participated in the study, which is not optimal, and more comprehensive studies are needed evaluating suicidal ideation in this population. Third, this study was conducted during the COVID-19 pandemic, a major confounding factor, and future studies in the nonpandemic situation are indicated. Fourth, we evaluated psychological and mental issues using self-administrated questionnaires; however, they might not be as accurate for evaluating these issues as an interview with an expert psychologist. Fifth, this study was conducted at TUMS, the best-known university in Iran, and students at TUMS may have different characteristics than students at other Iranian universities. Therefore, future multicenter studies in Iran may help in better evaluation of suicidal ideation in Iranian students. Sixth, we only evaluated students of three major healthcare fields, and our findings may not be applicable to other fields, especially those not related to healthcare and medicine. More studies are needed to evaluate the prevalence of suicidal ideation among Iranian students of other fields. Finally, we used convenience sampling methods in our study, which increases the risk of bias and chance error in this study, and future studies using random sampling methods are needed in the future. Despite these limitations, to the best of our knowledge, this was the first study evaluating the prevalence of suicidal ideation among Iranian pharmacy and dental students and comparing them with medical students, which can be a basis for further studies on the issue.

## 5. Conclusion

The prevalence of suicidal ideation among Iranian medical, dental, and pharmacy students is relatively high and has increased during recent years, requiring emergent action. Supportive and screening systems may be beneficial to provide early interventions for those at higher risk of suicidal ideation. Also, increasing the students, university officials, and educators' awareness about the prevalence of suicidal ideation and its associated factors can be another beneficial action in this regard. Those with a family history of psychological issues and separated parents are at a higher risk of having suicidal ideation, which should be noticed in screening programs. Smoking, hopelessness, loneliness, stress, and dissatisfaction with the current field were other factors that were independently associated with suicidal ideation in our study, and interventions for smoking cessation and improvements in curricula to reduce stress and increase their satisfaction with their fields may be beneficial to reduce the prevalence of suicidal ideation.

## Figures and Tables

**Table 1:** Family history and basic and demographic characteristics of participants.

Variable	Medical students (*N* = 133)	Pharmacy students (*N* = 85)	Dental students (*N* = 201)	*P* value	Without suicidal ideation	With suicidal ideation	*P* value
Gender							
Female	50 (37.6%)	50 (58.8%)	117 (58.2%)	<0.001	144 (50.5%)	73 (54.5%)	0.465
Male	83 (62.4%)	35 (41.2%)	84 (41.8%)		141 (49.5%)	61 (45.5%)	
Age (year)	24.15 (5.81)	21.35 (2.52)	21.89 (3.25)	0.001	22.31 (4.56)	22.88 (3.61)	0.006
Years at university							
1 year	22 (16.5%)	15 (17.6%)	40 (19.9%)	<0.001	54 (18.9%)	23 (17.2%)	0.014
2 years	36 (27.1%)	23 (27.1%)	38 (18.9%)		75 (26.3%)	22 (16.4%)	
3 years	26 (19.5%)	22 (25.9%)	14 (7%)		33 (11.6%)	29 (21.6%)	
4 years	17 (12.8%)	8 (9.4%)	34 (16.9%)		45 (15.8%)	14 (10.4%)	
5 years	10 (7.5%)	9 (10.6%)	25 (12.4%)		28 (9.8%)	16 (11.9%)	
6 years	4 (3%)	6 (6.1%)	35 (17.4%)		25 (8.8%)	20 (14.9%)	
7 years or more	18 (13.5%)	2 (2.4%)	15 (7.5%)		25 (8.8%)	10 (7.5%)	
Residence							
Home with family	67 (50.4%)	61 (71.8%)	124 (61.7%)	0.005	162 (56.8%)	90 (67.2%)	0.15
Home alone	17 (12.8%)	1 (1.2%)	12 (6%)		20 (7%)	10 (7.5%)	
Dormitory	45 (33.8%)	19 (22.4%)	61 (30.3%)		95 (33.3%)	30 (22.4%)	
Other	4 (3%)	4 (4.7%)	4 (2%)		8 (2.8%)	4 (3%)	
Marital status							
Single	112 (84.2%)	81 (95.3%)	178 (88.6%)	0.043	250 (87.7%)	121 (90.3%)	0.512
Married	21 (15.8%)	4 (4.7%)	23 (11.4%)		35 (12.3%)	13 (9.7%)	
Reason for choosing this field							
Family pressure	16 (12%)	3 (3.5%)	11 (5.5%)	<0.001	13 (4.6%)	17 (12.7%)	0.001
Social position	22 (16.5%)	10 (11.8%)	38 (18.9%)		49 (17.2%)	21 (15.7%)	
High salary	21 (15.8%)	11 (12.9%)	64 (31.8%)		58 (20.4%)	38 (28.4%)	
Personal interest	67 (50.4%)	52 (61.2%)	71 (35.3%)		145 (50.9%)	45 (33.6%)	
Other	7 (5.3%)	9 (10.6%)	17 (8.5%)		20 (7%)	13 (9.7%)	
Investigation about the field before choosing it							
No	37 (27.8%)	21 (24.7%)	34 (16.9%)	0.049	51 (17.9%)	41 (30.6%)	0.005
Yes	96 (72.2%)	64 (75.3%)	167 (83.1%)		234 (82.1%)	93 (69.4%)	
Father's age (year)	56.56 (7.45)	54.96 (4.99)	54.27 (5.26)	0.064	54.83 (6.12)	55.77 (5.94)	0.059
Mother's age (year)	51.17 (7.33)	49.78 (5.34)	49.06 (5.3)	0.074	49.42 (6.21)	50.65 (5.56)	0.006
Father's education							
Diploma or lower	27 (20.3%)	27 (31.8%)	48 (23.9%)	0.154	62 (21.8%)	40 (29.9%)	0.087
Higher than diploma	106 (79.7%)	58 (68.2%)	153 (76.1%)		223 (78.2%)	94 (70.1%)	
Mother's education							
Diploma or lower	42 (31.6%)	25 (29.4%)	72 (35.8%)	0.514	86 (30.2%)	53 (39.6%)	0.06
Higher than diploma	91 (68.4%)	60 (70.6%)	129 (64.2%)		199 (69.8%)	81 (60.4%)	
Parents living together							
Yes	125 (94%)	77 (90.6%)	192 (95.5%)	0.273	273 (95.8%)	121 (90.3%)	0.044
No	8 (6%)	8 (9.4%)	9 (4.5%)		12 (4.2%)	13 (9.7%)	
Father has passed away	4 (3%)	7 (8.2%)	5 (2.5%)	0.057	11 (3.9%)	5 (3.7%)	1
Mother has passed away	3 (2.3%)	0 (0%)	1 (0.05%)	0.162	2 (0.7%)	2 (1.5%)	0.596
Family income per month							
< 5 million Toman	4 (3%)	15 (17.6%)	25 (12.4%)	<0.001	24 (8.4%)	20 (14.9%)	0.323
5-9.99 million Toman	32 (24.1%)	24 (28.2%)	65 (32.3%)		87 (30.5%)	34 (25.4%)	
10-14.99 million Toman	44 (33.1%)	21 (24.7%)	36 (17.9%)		70 (24.6%)	31 (23.1%)	
15-19.99 million Toman	17 (12.8%)	9 (10.6%)	34 (16.9%)		40 (14%)	20 (14.9%)	
≥ 20 million Toman	36 (27.1%)	16 (18.8%)	41 (20.4%)		64 (22.5%)	29 (21.6%)	
Family history of psychological issues	19 (14.3%)	15 (17.6%)	24 (11.9%)	0.435	27 (9.5%)	31 (23.1%)	<0.001
Family history of self-harm	7 (5.3%)	4 (4.7%)	6 (3%)	0.554	8 (2.8%)	9 (6.7%)	0.067
Family history of suicide attempt	4 (3%)	4 (4.7%)	6 (3%)	0.735	11 (3.9%)	3 (2.2%)	0.563
Smoking							
Never	121 (91%)	68 (80%)	165 (82.1%)	0.09	255 (89.5%)	99 (73.9%)	<0.001
Quite	3 (2.3%)	2 (2.4%)	9 (4.5%)		10 (3.5%)	4 (3%)	
Yes	9 (6.8%)	15 (17.6%)	27 (13.4%)		20 (7%)	31 (23.1%)	
Addiction							
Never	131 (98.5%)	84 (98.8%)	192 (95.5%)	0.272	281 (98.6%)	126 (94%)	0.007
Quite	0 (0%)	0 (0%)	4 (2%)		0 (0%)	4 (3%)	
Yes	2 (1.5%)	1 (1.2%)	5 (2.5%)		4 (1.4%)	4 (3%)	
Family history of smoking							
No	93 (69.9%)	59 (69.4%)	146 (72.6%)	0.803	202 (70.9%)	96 (71.6%)	0.908
Yes	40 (30.1%)	26 (30.6%)	55 (27.4%)		83 (29.1%)	38 (28.4%)	
Family history of addiction							
No	125 (94%)	78 (91.8%)	188 (93.5%)	0.803	267 (93.7%)	124 (92.5%)	0.678
Yes	8 (6%)	7 (8.2%)	13 (6.5%)		18 (6.3%)	10 (7.5%)	
Satisfaction with current field	3 (1)	4 (1)	4 (2)	0.003	4 (1.5)	3 (2)	<0.001

Values are reported as number (percentage), except for age, father's age, and mother's age that are reported as mean (SD), and satisfaction with the current field that is reported as median (IQR).

**Table 2:** Comparing the psychological issues, suicidal ideation, and suicidal attempt among medical, dental, and pharmacy students.

	Medical student (*N* = 133)	Pharmacy student (*N* = 85)	Dental student (*N* = 201)	*P* value	Without suicidal ideation	With suicidal ideation	*P* value
BHS	7.28 (4.72)	7.8 (4.59)	5.9 (4.17)	<0.001	5.2 (3.68)	9.97 (4.39)	<0.001
PSS	28.19 (8.65)	29.69 (8.84)	26.91 (7.94)	0.069	25.11 (7.18)	33.76 (7.8)	<0.001
UCLA loneliness scale	45.45 (13.42)	49.3 (12.95)	44.23 (12.57)	0.01	41.57 (11.53)	54.31 (11.78)	<0.001
MBI-SS-exhaustion	13.9 (7.06)	14.77 (6.82)	13.65 (6.96)	0.4	12.7 (6.57)	16.62 (7.03)	<0.001
MBI-SS-cynicism	10.87 (5.97)	12.85 (6.39)	11.01 (5.89)	0.034	10.22 (5.82)	13.73 (5.86)	<0.001
MBI-SS-professional efficacy	17.72 (7.47)	16.75 (7.49)	17.86 (7.48)	0.453	19.08 (7.07)	14.43 (7.35)	<0.001
History of psychological issues	17 (12.8%)	19 (22.4%)	23 (11.4%)	0.046	19 (6.7%)	40 (29.9%)	<0.001
History of self-harm	7 (5.3%)	12 (14.1%)	15 (7.5%)	0.059	18 (6.3%)	16 (11.9%)	0.056
History of suicide attempt	2 (1.5%)	7 (8.2%)	9 (4.5%)	0.057	8 (2.8%)	10 (7.5%)	0.038
Suicidal ideation	45 (33.8%)	29 (34.1%)	60 (29.9%)	0.668	—	—	—

Values are reported as mean (SD), except for history of psychological issues, history of self-harm, suicidal ideation, and history of suicide attempt that are reported as number (percentage). BHS: Beck Hopelessness Scale; PSS: Perceived Stress Scale; MBI-SS: Maslach Burnout Inventory-Student Survey.

**Table 3:** Results of the multiple binary logistic regressions determining the factors associated with suicidal ideation among healthcare-profession students.

Independent variable	OR	95% CI	*P* value
Family history, basic and demographic characteristics			
Family history of psychological issues	2.186	1.184-4.036	0.012
Current or past smoking	2.155	1.197-3.881	0.01
Parents not living together	2.512	1.018-6-199	0.046
Satisfaction with current field	0.51	0.41-0.634	<0.001
Questionnaires' scores			
BHS	1.167	1.083-1.258	<0.001
PSS	1.081	1.032-1.132	0.001
UCLA loneliness scale	1.057	1.032-1.084	<0.001

The presence of suicidal ideation was the dependent variable.

## Data Availability

The data that support the findings of this study are available from the corresponding author, MHS, upon reasonable request.
